# Case Report: Metagenomic Next-Generation Sequencing Confirmed a Case of Central Nervous System Infection With *Brucella melitensis* in Non-endemic Areas

**DOI:** 10.3389/fmed.2021.723197

**Published:** 2021-09-14

**Authors:** Jun Cao, Qingqing Cai, Wentao Su, Zi Ge, Hui Zhao, Xinjian Zhou, Ke Ma, Zhijie Xia

**Affiliations:** ^1^Department of Emergency and Critical Care Medicine, Fudan University Affiliated North Huashan Hospital, Shanghai, China; ^2^Genoxor Medical Science and Technology Inc., Shanghai, China

**Keywords:** metagenomic next-generation sequencing, *Brucella melitensis*, doxycycline, rifampin, central nervous system infection

## Abstract

Brucellosis is a highly contagious zoonotic disease caused by bacteria that belong to the genus *Brucella*. It is a major endemic disease in northern China. We reported a rare case of central nervous system (CNS) infection caused by *Brucella melitensis* in a patient living in non-endemic areas. The medical history of the patient included chronic headache and trunk numbness. Based on the presented clinical symptoms and medical examinations, a clinical diagnosis of binocular uveo-encephalitis was made in the local hospital. The patient's symptoms were unrelieved after being treated with empiric therapy. Soon after, the patient was admitted to our hospital because of the obnubilation and coma in the trip. We ran a few examinations and sent the cerebrospinal fluid (CSF) for metagenomic next-generation sequencing (mNGS) immediately. The Magnetic resonance imaging (MRI) examination was unremarkable, and bilateral mastoid inflammation was attached. Metagenomic next-generation sequencing suggested a CNS infection caused by *Brucella melitensis*. Then, the results of the serum agglutination test and quantitative polymerase chain reaction assay also confirmed that. After being treated with doxycycline, rifampin, and cefatriaxone, consciousness of the patient was restored and headache diminished. Two months later, a lumbar puncture was used to check the pressure of the CSF, and the total course of treatment was more than 6 months. This case highlighted the potential value of mNGS in early clinal diagnosis. We believe that mNGS may be a complementary method for rapid identification of infection of CNS caused by the pathogen.

## Introduction

*Brucella* species are small Gram negative, non-motile, non-spore-bearing coccobacilli, which cause chronic disease both in humans and animals (1, 2). In all species, *Brucella melitensis* has been the main pathogenic strain associated with human brucellosis in China. According to the surveillance data confirmed by the Centers for Disease Control and Prevention (CDC) from Inner Mongolia (northern province of China), a total of 43,623 cases were reported during 1999–2008 (3, 4). The clinical manifestations of human brucellosis vary dramatically, which can vary from asymptomatic infection to systemic infection, such as hepatic, cardiac, ocular, or central nervous system (CNS) (5). Given the high mortality of CNS infection, accurate diagnosis and appropriate therapy could provide patients with favorable prognosis. In this report, we described a male patient in the non-endemic area with atypical symptoms such as fever and nervous signs. Metagenomic next-generation sequencing (mNGS) was used in the early diagnosis of neurological brucellosis caused by *Brucella melitensis*.

## Case Report

A 39-year-old male patient with uveitis caused by the infection of two eyes was initially presented to the local hospital in September 2020 after having paralysis of both the lower limbs and blurred vision for 30 days. He was administered medicine for invigorating blood circulation and eliminating stasis, glucocorticoid, and nutrition treatment for 10 days. On October 10, 2020, he was discharged from the hospital after his headache and the symptom of the paralysis of lower limbs diminished. However, he continued to have the symptom of blurred vision and was treated with 40 mg prednisone every day.

Three days later, the patient's headache was aggravated, and he suddenly developed symptoms such as obnubilation, tic of limbs, foaming at the mouth, and fever up to 38.0°C during his trip to Shanghai on October 14, 2020. After an emergency treatment, he was presented to our hospital the next day. On admission, he was still suffering from obnubilation and restlessness. Then, he was immediately given mechanical ventilation and infused with remifentanil and propofol 6 mL/h *via* an intravenous pump. Medical history of the patient was reported later, which included a 2-month history of recurrent headache, dizziness, and nausea without obvious inducement, accompanied by intermittent fever, and the maximum body temperature was 38.0°C which could be resolved by ibuprofen. Some imaging examinations were ordered in September 2020 by his primary care physician to diagnose the main cause of the headache. The results of magnetic resonance angiography of cerebral vascular showed right fetal posterior cerebral arteries. Transcranial Doppler showed quick blood velocity of the vertebrobasilar artery and bilateral posterior cerebral arteries, but no remarkable abnormality of his brain was found in MRI. Consequently, the patient was preliminarily diagnosed as infection of CNS combined with secondary epilepsy and acute respiratory failure.

After admission, routine laboratory examinations and mNGS (Genoxor Medical Science and Technology Inc., Shanghai, China) for the cerebrospinal fluid (CSF) were performed on a Nextseq 550 platform that was ordered concurrently to inspect possible etiology. The maximum body temperature increased to 38.6°C. The leukocyte count in peripheral blood was 7.25 × 10^9^/L, of which neutrophils accounted for 80.5%. The level of C-reactive protein was elevated to 66.79 mg/L, fungal (1, 3)-β-D-glucan detections (G test) increased to 150.7 pg/mL, and CD64 infection index was 3.4. Serum electrolyte indicated the disorder of sodium and chloride. The functions of the liver and thyroid were basically normal, moreover, the indicators of autoimmune diseases were negative. In addition, IgM antibodies tests of nine kinds of respiratory pathogens including *Legionella pneumophila, Mycoplasma pneumonia, Rickettsia of Q fever, Chlamydia pneumoniae, Adenovirus, Respiratory syncytial virus, Influenza A virus, Influenza B virus*, and *Parainfluenza virus* were negative, simultaneously, the results of sputum Gram stain and culture were negative too. The diagnostic workup for infectious diseases was unrevealing. However, the result of mNGS revealed 199 reads of *Brucella* genus (a total of 21,542,240 sequence reads), 4 unique reads corresponding to *Brucella melitensis* in the cerebrospinal fluid within 24 h. We have detected cerebrospinal fluid of the patient by quantitative polymerase chain reaction (Supplementary Figure 1) with specific primers (Forward primer: GCCAAATATCCATGCGGGAAG, reverse primer: TGGGCATTCTCTACGGTG) for *Brucella melitensis* we designed, which indicated infection of *Brucella melitensis*. After reconfirming the historical experience of the patient, which included contact with a sick goat was known. The patient was rediagnosed as infection of CNS was caused by *Brucella melitensis*, which was combined with secondary epilepsy. Consequently, doxycycline (0.1 g q12h), sulfamethoxazole (2# q12h), and amoxicillin-clavulanic acid (2.4 g q12h) were administrated for initial treatment of anti-infection, combined with mannitol applied to reduce the intracranial pressure. After 4 days of treatment, the patient gradually regained consciousness and recovered to spontaneous respiration and oral feeding.

**Figure 1:**
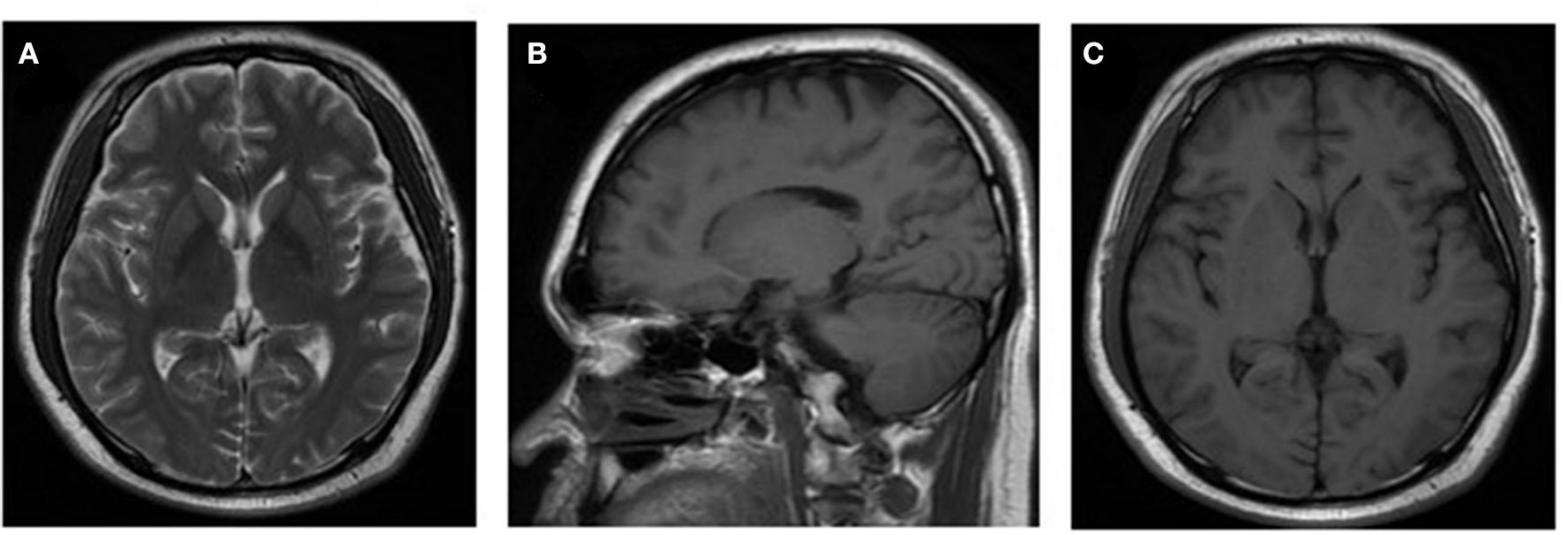
Neuroradiologic MRI. The MRI examination results of the patient were unremarkable (images shown in pictures **A–C**).

**Figure 2:**
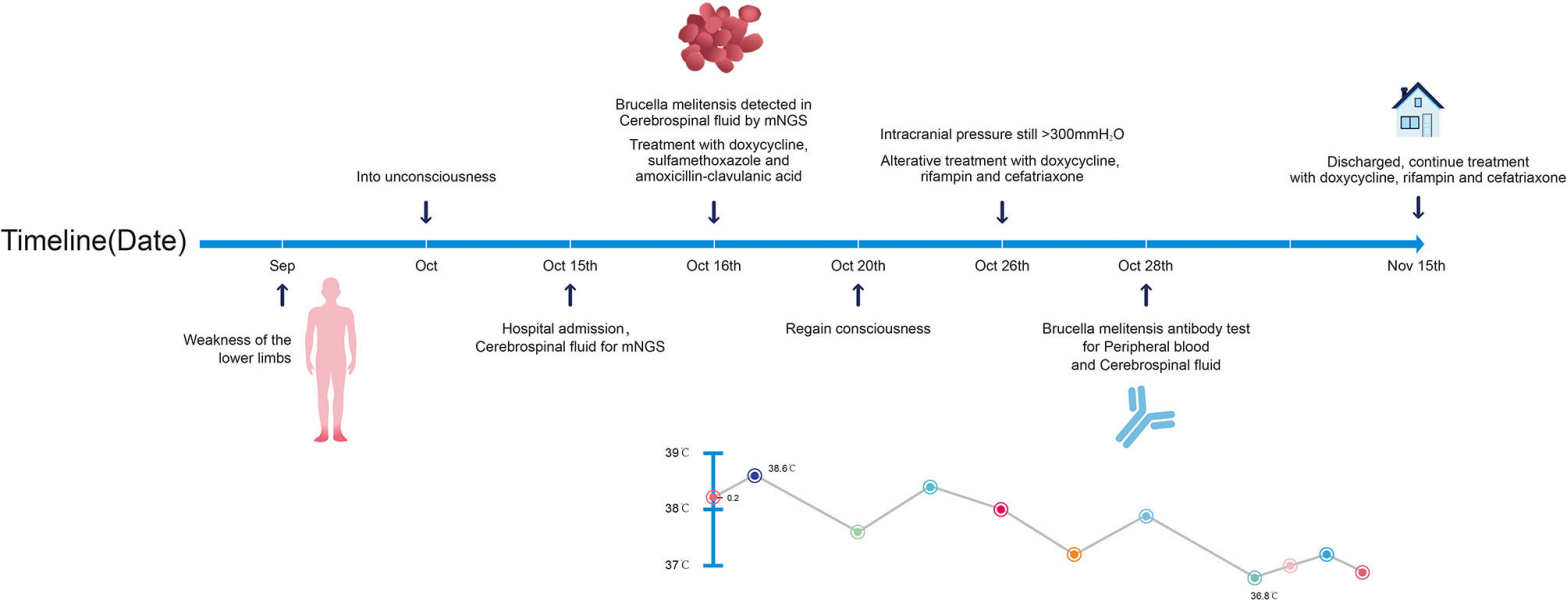
Timeline of the case report.

However, after 10 days of initial treatment, the review of CSF pressure revealed that the intracranial pressure was still > 300 mmH_2_O. On suspicion of unknown etiology, both peripheral blood and CSF samples of the patient were sent for mNGS, combined with CSF examined by traditional examinations too. The karyocyte count of CFS was 210 × 10^6^/L (60% monocyte and 40% apocyte), erythrocyte count was 360 × 10^6^/L, with a glucose level of 5.29 mmol/L. The protein level of cerebrospinal fluid was 4.39 g/L, the lactic level was 5.4 mmol/L, and the chloride level decreased to 105 mmol/L. Two sets of cultures of CSF, and also the test for *EB virus* antigen, *Human cytomegalovirus* antibody, *Herpes simplex virus* antibody, *Cryptococcus* antigen, and *Toxoplasma* antibody, were negative (Full laboratory testing in CSF is shown in Table 1). In addition, the results of echocardiography, vascular ultrasound, and MRI of the head were all unremarkable (Figure 1). However, within 24 h, mNGS detected 22 reads (A total of 19,870,653 sequence reads) corresponding to *Brucella* genus in CSF, but nothing was tested in peripheral blood. Subsequently, the identification of *Brucella* was confirmed by the SAT kit (Sinoreca, Beijing) in CSF and peripheral blood, antibody titers against *Brucella* in CSF and peripheral blood were 1:80 and 1:160, respectively. Therefore, the treatment was adjusted to doxycycline (0.1 g q8h), rifampin (0.45 g qd), and cefatriaxone (2.0 g ivgtt bid). After 20 consecutive days of the new treatment, his headache and blurred vision diminished, with body temperature returning to normal. The patient was discharged from the hospital with cefatriaxone (2.0 g ivgtt bid), doxycycline (0.1 g tid), and rifampicin (0.15 g tid) (The timeline of this case can be seen in Figure 2). About 2 months later, the patient returned to Shanghai for a lumbar puncture, and the pressure of cerebrospinal fluid was 210 mmH_2_O. The follow-up to maintain the oral anti-infective program, the total course of the treatment is about 6 months, symptoms of the patient have basically disappeared, the body temperature was stable, and antibiotics were stopped.

**Table 1:** The data of diagnostic workup for the cerebrospinal fluid (CSF) sample after 10 days of initial treatment.

**Parameter (unit)**	**Result**	**Normal range**
Karyocyte (10^6^/L)	210	N/A
Red cell (10^6^/L)	360	N/A
CSF protein (g/L)	4.39	0.15–0.45
Glucose (mmol/L)	5.29	2.22–3.78
Lactate (mmol/L)	5.4	1.1–2.4
Chloride(mmol/L)	105	120–132
Pandy test	+++	N/A
IgM of CMV, HSV, EBV, Toxoplasma	Negative	N/A
IgG of CMV	23.4	<12.0 U/mL, Negative
IgG of HSV	Negative	N/A
IgG of EBVCA, EBVNA, EBVEA	<10,16.4, <5	<12.0 U/mL, Negative
IgG of Toxoplasma	Negative	N/A
Ink staining	Negative	N/A
IgM, IgG of Cryptococcus	Negative	N/A

*CMV, cytomegalovirus; HSV, herpes simplex virus; EBV, Epstein-Barr virus; EBVNA, Epstein-barr virus nuclear antigen; EBVCA, Epstein-Barr Virus capsid Antigen; EBVEA, Epstein-Barr virus early antigen; N/A, not applicable*.

## Discussion

Brucellosis is a widespread zoonosis caused by pathogenic *Brucella*, a group of Gram-negative facultative intracellular bacteria (6, 7). Although the main hosts of *Brucella* are sheep, cattle, swine, and goat, it can be transmitted to humans when the contaminated milk, dairy products, and meat are ingested, or when individuals are in contact with infected animals (8). In the recent years, 12 species of *Brucella* are isolated both from marine and land mammals (2, 7, 9), four of them have the zoonotic characteristic, the most virulent species that can cause human brucellosis is *B. melitensis*, followed successively by *B. abortus, B. suis*, and *B. canis* (1). In this case, the patient was infected by *B. melitensis* after contacting with infected sheep and suffered from CNS infection with multiple symptoms.

*Brucella* species can cause systemic disease in humans, especially damages to the reproductive system and joints of the individuals (10, 11). However, the incidence of neurological involvement is rare, with about 2 to 5% of patients involved with the nervous system. Neurobrucellosis (NB) is the most serious complication of brucellosis, which can involve the CNS and the peripheral nervous system (12). The main clinical presentations of the patients include meningitis, encephalitis, meningoencephalitis, radiculitis, myelitis, optic neuritis, and behavioral abnormalities, among which meningitis is the most common presentation accounting for 17 to 74% of NB (5, 13). Although the involvement of CNS is rare, it severely restricts the survival rate of the patients without accurate diagnosis and timely treatment. In this case, the patient was presented to the local ophthalmic department with uveitis and was treated for 10 days. He was admitted to our hospital 1 month before. Three days after the patient was discharged from the ophthalmic department, his headache was aggravated, and he suddenly developed symptoms, such as obnubilation, tic of limbs, foaming at the mouth, with a fever up to 38.0°C. In addition, his symptom of blurred vision was not disappeared until the use of the antibrucellosis drug. Therefore, we suspected that *B. melitensis* may be the cause of uveitis and was not diagnosed in an accurate and time manner.

Although infection of *Brucellar* can be diagnosed by culture, serological test, and PCR-based tests, culture remains to be the gold standard for diagnosis (14). Actually, clinical microbiology laboratory culture for detecting *Brucella* of acute cases usually takes at least 5 to 7 days with a modern automated blood culture system, and protracted ones may need a longer time to obtain a definitive result (8, 14). The long incubation and low sensitivity hamper the detection of cultures and cause the delay in diagnosis, furthermore, the safety of the laboratory is still a worldwide concern. Of course, the serological test is another widely used pathogen diagnostic method of human brucellosis that has drawbacks too, such as insufficient specificity and poor repeatability. In recent years, nucleic acid amplification tests have been developed into a new tool with excellent sensitivity and specificity, and regarded as supplemental support to culture, nevertheless, it is limited to the known gene sequence of pathogenic microorganisms and remains have high false positivity in the recovered patients. Therefore, the selection of an appropriate pathogen diagnostic method is essential for the diagnosis of *brucellosis*, especially in patients with CNS involvement. In this case, consciousness of the patient was lost and was in poor health status upon admission, and the MRI examination results of the patient were unremarkable, which made it more difficult for us to judge the central infection. We decided to send the CSF sample for mNGS directly without waiting for the culture of it. After a trade-off analysis between the benefits of treatment and the risk of false discovery of *B. melitensis*, initial treatment was made immediately based on the result of mNGS before confirmatory tests were completed. The patient was treated with doxycycline, compound sulfamethoxazole tablets, and amoxicillin-clavulanic acid for anti-infection as an initial treatment. The treatment effect was valuated from the number of unique reads in the CSF sample of October 16, 2020 and October 26, 2020, after 10 days of treatment the number of *B. melitensis* unique reads decreased to 0 and the number of *Brucella* unique reads decreased from 199 to 22. More and more cases suggested that mNGS could be implemented for monitoring the progress of the disease and evaluating therapy effects. Furthermore, the multiple symptoms of the patient were relieved. Due to *Brucella* was not the main cause of CNS infection, and the atypical clinical presentations of the patient, tended to be misdiagnosed or inappropriately treated. This case showed that using mNGS for inspecting the possible etiology of CNS infection was the right choice.

However, the review of pressure of CSF revealed that there was still intracranial infection. The concern of unknown pathogenic infection was denied due to the result of mNGS showed that there were only 22 reads of *Brucella* with no new etiology or *B. melitensis*. Therefore, we replaced doxycycline, rifampin, and cefatriaxone for anti-infection treatment. After a continued new treatment of 20 days, the condition of the patient was gradually stabilized and discharged. This was a rare case of CNS infection with *B. melitensis*, which lasted for days after the acute exacerbation. Although mNGS has some limitations such as contamination of human genome and environmental microorganisms, the development of depletion methods and targeted sequencing are overcoming these defects (15). In this case, the CSF samples of the patient were detected by mNGS at two critical time points, and the targeted antimicrobial therapy was attributed to the discovery of mNGS.

In summary, our case indicated that mNGS was a useful diagnostic tool to detect causative pathogen of CNS infection without typical presentations, especially for the guidance of clinical medication.

## Data Availability Statement

The datasets presented in this study can be found in online repositories: The data that support the findings of this study are openly available in NCBI with accession number SRR14750292, SRR14750291, SRR14750290, and SRR14744775.

## Ethics Statement

Written informed consent was obtained from the individual(s) for the publication of any potentially identifiable images or data included in this article.

## Author Contributions

ZX and JC analyzed and explained the data and drafted and revised the manuscript. QC and WS designed the study. HZ, KM, and XZ acquired data. ZX and JC revised the manuscript. All authors approved the final manuscript.

## Funding

This study was supported by the Special Project of Shanghai Municipal Economic and Information Technology Commission (Project No. 201601028), the Key Medical Project of Science, the Technology Support Plan of Shanghai Science and Technology Commission (Project No. 1641 1954400), and the General Project of Shanghai Municipal Health and Family Planning Commission (Project No. 201640181).

## Conflict of Interest

QC is employed by Genoxor Medical Science and Technology Inc. The remaining authors declare that the research was conducted in the absence of any commercial or financial relationships that could be construed as a potential interest conflict of interest.

## Publisher's Note

All claims expressed in this article are solely those of the authors and do not necessarily represent those of their affiliated organizations, or those of the publisher, the editors and the reviewers. Any product that may be evaluated in this article, or claim that may be made by its manufacturer, is not guaranteed or endorsed by the publisher.

## Supplementary Material

Supplementary Figure 1 Analysis of Quantitative polymerase chain reaction (qPCR) the *Brucella melitensis*. Amplification curve of the qPCR confirmed the *Brucella melitensis*.
