# Supplementary figures and images for: Case Report: Metagenomic Next-Generation Sequencing Confirmed a Case of Central Nervous System Infection With Brucella melitensis in Non-endemic Areas
Source: Front Med (Lausanne). 2021 Sep 14;8:723197. doi: 10.3389/fmed.2021.723197 (PMC8476800; doi:10.3389/fmed.2021.723197)

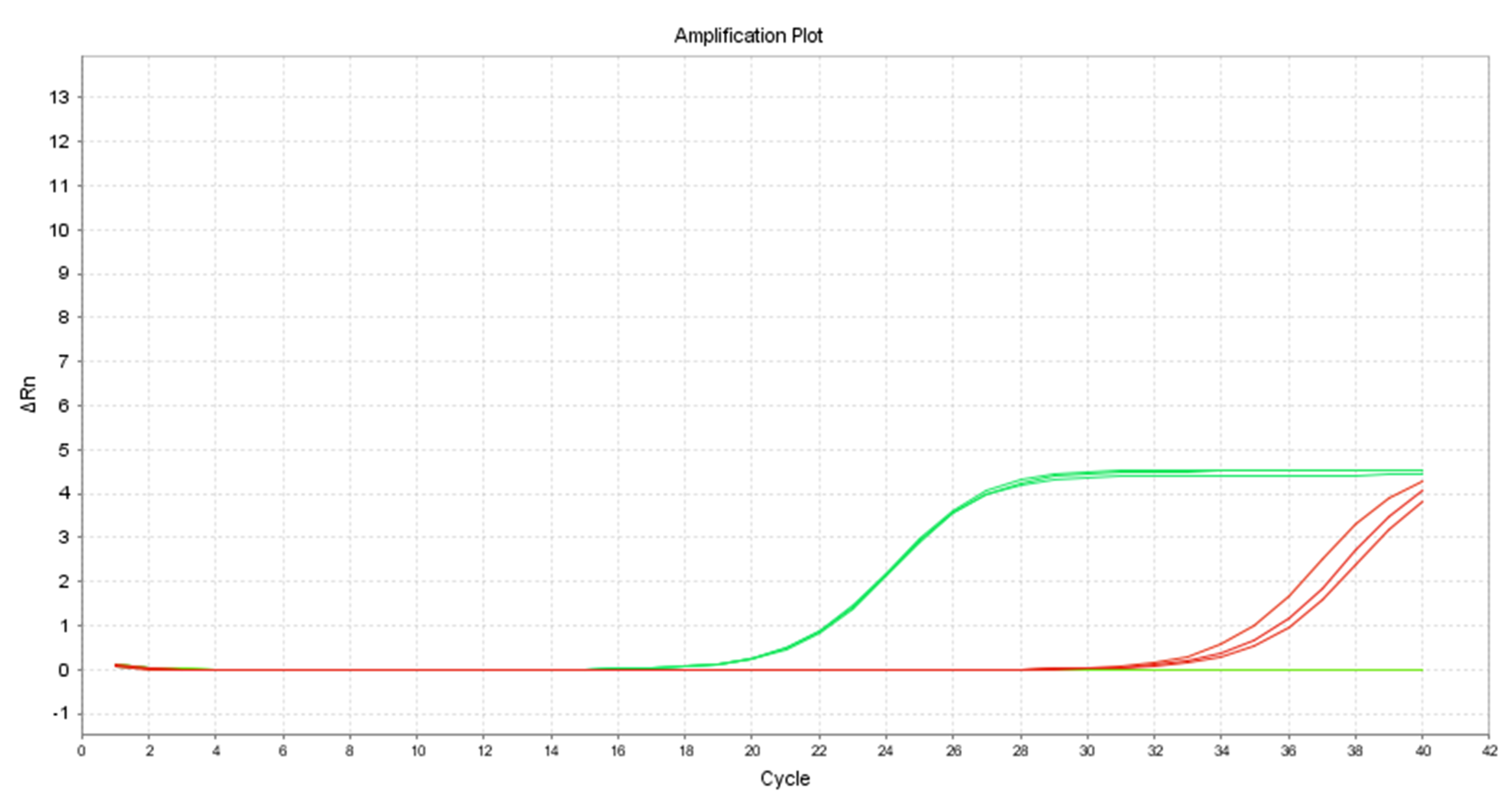

Supplement: Supplementary Figure 1 — Analysis of Quantitative polymerase chain reaction (qPCR) the Brucella melitensis. Amplification curve of the qPCR confirmed the Brucella melitensis. [file Image_1.png]
