# Supplementary material for: Case Report: Metagenomic Next-Generation Sequencing Confirmed a Case of Central Nervous System Infection With Brucella melitensis in Non-endemic Areas
Source: Front Med (Lausanne). 2021 Sep 14;8:723197. doi: 10.3389/fmed.2021.723197 (PMC8476800; doi:10.3389/fmed.2021.723197)
Supplement: Supplementary file 2 [file Table_1.DOCX]

Supplementary materials

**mNGS workflow**

mNGS of specimen DNA was extracted using QIAamp DNeasy Blood & Tissue Kit (Qiagen). The quantity and quality of DNA was assesses using the Qubit (Thermo Fisher Scientific) and NanoDrop (Thermo Fisher Scientific), respectively. DNA libraries were constructed by fragmenting DNA into 200 300 bp, which were flat end-repaired and barcode adapter-ligated. The sequencing library was constructed via KAPA BIOSYSTEM library Prep Kit KK8504. The quality control of the DNA libraries was analyzed with Agilent 2100 equipment (Agilent Technologies). The library was 75bp single-end sequenced by Illumina NextSeq system. The raw data were generated by Illumina sequencing machine (NextSeq 550Dx). High-quality data were generated after filtering out adapter, low-quality, low-complexity, and shorter reads (length<36 bp). Human host sequence were identified by mapping to human reference genome (hs37d5) using bowtie2 software. The remaining data were aligned to the microbial genome database, which is more than 20000 genome. Our microorganism genome database contained bacteria, fungi, virus and parasite genomic sequences (download from ftp://ftp.ncbi.nlm.nih.gov/genomes/genbank/). Finally, we got the microbial compositions of the sample.
